# Scientific and Regulatory Lessons Learnt on Building a Chemistry, Manufacturing, and Controls (CMC) Package for COVID-19 Variant Vaccine Updates in the EU—A Regulator’s Perspective

**DOI:** 10.3390/vaccines12111234

**Published:** 2024-10-29

**Authors:** Ragini Shivji, Elena Grabski, Veronika Jekerle

**Affiliations:** 1 European Medicines Agency, Human Division, Domenico Scarlattilaan 6, 1083 HS Amsterdam, The Netherlands; veronika.jekerle@ema.europa.eu; 2 Division of Infectology, Paul-Ehrlich-Institut, Federal Institute for Vaccines and Biomedicines, Paul-Ehrlich-Straße 51-59, 63225 Langen, Germany; elena.grabski@pei.de

**Keywords:** COVID-19 vaccine, chemistry, manufacturing, and controls, regulatory approvals, variant updates

## Abstract

During the COVID-19 pandemic, eight COVID-19 vaccines were authorised in the European Union (EU); as a result of emerging SARS-CoV-2 variants and waning immunity, some of these have been adapted to broaden the immunity against circulating variants. The pace at which variants emerge challenges the technical feasibility to make adapted vaccines available in a suitable timeframe and in sufficient quantities. Despite the current absence of a clear-cut seasonal spread for COVID-19, the EU regulatory approach thus far is a pragmatic approach following a pathway similar to that of seasonal influenza. This approach currently requires chemistry, manufacturing, and controls (CMC—the design, development and consistent manufacture of a specified medicinal product of good quality) and non-clinical data (from product laboratory and animal studies), as well as demonstrating that updated vaccines induce an immune response that can predict clinical efficacy and safety in humans. For CMC data, COVID-19 mRNA vaccine adaptations generally made use of the same formulation, control strategy, manufacturing process, and inclusion of registered manufacturing sites for the drug product; therefore assessment was generally streamlined. The experience gained from the vaccine adaptations, combined with a continuous early regulator-developer scientific discussion, permits increasingly greater predictability for timing and positive regulatory outcomes. Here, we review key aspects of the quality control and manufacture of updating COVID-19 vaccines to protect against new variants. Although most experience has been gained with mRNA vaccines, we note that investment in the streamlining of manufacturing processes for recombinant protein vaccines would facilitate future strain updates/adaptations thereby safeguarding availability of different COVID-19 vaccine types, which is considered of value for public health. We also reflect on the challenges and opportunities in establishing more predictable regulatory mechanisms for future COVID-19 vaccine adaptions and more widely for future vaccines containing rapidly evolving pathogens with the potential to cause health threats.

## 1. Introduction

During the COVID-19 pandemic eight COVID-19 vaccines have been authorised in the EU [1,2] (some MAs now withdrawn due to commercial reasons). Based on public health needs, given waning population immunity and viral evolution, some vaccines were subsequently adapted to emerging COVID-19 variants, which were immunologically distant from the original (ancestral) SARS-CoV-2 strain approved [3]. A mechanism for adaptation of the vaccines targeting the original ancestral strain was established.

In the EU in 2022, only the mRNA vaccines were adapted (to Omicron BA.1, then Omicron BA.4-5). In 2023, adaptations were made to two mRNA vaccines and one recombinant protein vaccine (all to Omicron XBB.1.5). The current regulatory strategy for these products, which have now accumulated variant update experience, requires CMC and non-clinical data, as well as demonstrating that updated vaccines induce an immune response that can predict clinical efficacy and safety in humans [4]. These updates intend to broaden immunity against circulating variants and thus support vaccination strategies, as evidenced by clinical studies with the adapted vaccines [5,6,7,8,9]. The European Medicines Agency (EMA) collaborates with international medicines regulators and the World Health Organization (WHO) to align strain recommendations as far as possible [10].

The CMC section of the marketing authorisation (MA) is driven by the clinical requirements, and the EMA’s regulatory decisions are taken in the context of benefit–risk, i.e., medicines are authorised only when their benefits outweigh their risks for the target population. This regulatory evaluation is independent of both cost considerations for developers or purchasers, and decisions on whether and which vaccine to use in vaccination campaigns, which is within the remit of EU member states. Here, we communicate our experience of the CMC part of applications to update/adapt the COVID-19 vaccines, these updates being based on recommendations from the EMA’s Emergency Task Force (ETF) [11]. Lessons learnt from the EMA’s scientific assessments can inform more predictable, robust applications and facilitate approval, supply and immunisation of adapted COVID-19 vaccines and also provide a good basis from which to reflect on future requirements for other vaccines for public health emergencies.

## 2. Vaccine Authorisations and Evolving Epidemiology of SARS-CoV-2

The first COVID-19 vaccine was authorised in the EU on 21 December 2020 (Comirnaty) [12]. Spikevax [13] was subsequently authorised on 06 January 2021. Both vaccines made use of mRNA technology, which in addition to its novelty proved versatile for rapid vaccine development.

Two further products were authorised subsequently, viral-vectored vaccines Vaxzevria [14], (authorised 29 January 2021, now withdrawn) and Jcovden [15] (authorised on 11 March 2021). The next product (COVID-19 vaccine Valneva [16], authorised and subsequently withdrawn) was an inactivated, adjuvanted vaccine. The first recombinant protein vaccine, Nuvaxovid [17], was authorised on 20 December 2021.

All these initially authorised vaccines contained constructs targeting the SARS-CoV-2 ancestral strain. However, global epidemiological surveillance identified the rapid evolution of the SARS-CoV-2 virus in the human population and numerous variants were identified [18]. By the end of 2020, various variants of concern (VOC) had emerged, including Alpha (B.1.1.7), Beta (B.1.351), and Gamma (B.1.1.28) [19]. During 2021, Delta (B.1.617.2) became the dominant variant worldwide [20], which was then replaced by Omicron sub-lineages, BA.1 by early 2022 [21] and eventually by Omicron BA.4/BA.5 during 2022 [22]. By June 2023, the XBB.1 descendent lineages dominated globally [3].

In light of waning protection over time, companies and regulators recognised early on that changes to the authorised vaccines were needed to maintain vaccine effectiveness against circulating SARS-CoV-2 strains.

In the EU, the ETF [11] is responsible for recommending updates to antigenic composition of COVID-19 vaccines. Both authorised mRNA vaccines, Comirnaty (Comirnaty is a bivalent presentation containing the ancestral original and Omicron BA.1 approved 1 September 2022 and a bivalent presentation containing the ancestral original and Omicron BA.4-5 approved 12 September 2022.) and Spikevax (Spikevax is a bivalent presentation containing the ancestral original and Omicron BA.1 approved 1 September 2022 and a bivalent presentation containing the ancestral Original and Omicron BA.4-5 approved 20 October 2022) were updated in 2022. In the context of the epidemiological situation, the manufacturer of VidPrevytn Beta [23] (MA now withdrawn) changed the drug substance of this adjuvanted recombinant protein vaccine to a version of the spike protein found on the surface of SARS-CoV-2 Beta variant. VidPrevytn Beta was authorised in 2022 followed by Bimervax, an adjuvanted recombinant protein vaccine, containing a SARS-CoV-2 receptor-binding domain fusion heterodimer of B.1.351 (Beta)–B.1.1.7 (alpha) strains, in 2023.

**Figure 1:**
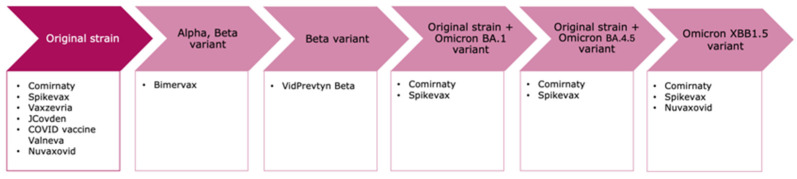
Previously EU authorised COVID-19 vaccines and the corresponding targeted SARS-CoV-2 strains *. * Information correct as of 19 June 24. Note that not all vaccines nor all variant presentations may still be authorised [2].

Available data confirmed that approved COVID-19 vaccines, including those based on the ancestral index virus, continued to provide protection against severe disease even in 2023. However, protection declined as the virus mutated to immunologically distant variants from strains included in the vaccines [3]. In preparation of the autumn/winter season of 2023, variant updates to the mRNA vaccines (monovalent vaccines) were approved (Comirnaty XBB.1.5 approved 31 August 2023; Spikevax XBB.1.5 approved 15 September 2023) and, subsequently, the first update to a recombinant protein vaccine was approved (Nuvaxovid XBB.1.5 approved 31 October 2023). See Figure 1 for information on all previously authorised EU SARS-CoV-2 vaccines.

## 3. Updates of Existing Marketing Authorisation (MA)—Legal Framework and Regulatory Considerations

The existing regulatory framework to support evaluation of a medicinal product [24,25] has been challenged with respect to evaluation timelines during the COVID-19 pandemic. Although coordinated EU actions have benefited all EU member states in terms of MA approvals and vaccine supply, the requirement for specific preparedness and response tools has also been emphasised [26].

In 2021, during the emergency phase of the pandemic, legislative changes permitted change of the drug substance for a human coronavirus vaccine by a regulatory change (Type II variation) to the MA. This is a fast-track procedure well established for the annual update of seasonal influenza vaccines and avoids the lengthier process of having to apply for a full marketing authorisation.

Unlike the influenza provisions, where the drug substance is replaced each season, for coronavirus, to acknowledge the absence of seasonality thus far, the legal provisions permit addition of new drug substances/combinations of drug substances within the same MA. Indeed, in the first round of COVID-19 mRNA vaccine MA updates to Comirnaty and Spikevax in 2022, the resulting products were bivalent presentations containing the SARS-CoV-2 ancestral strain and Omicron BA.1 and, subsequently, the SARS-CoV-2 ancestral strain and Omicron BA.4-5. For EU-authorised influenza vaccines, only changes related to the new influenza strains used may be introduced via the ‘fast track’ procedure [27,28] to ensure that the adapted review timelines can be consistently achieved. This permits update of the marketing authorisation with quality data related to the new strain and a requirement for post-authorisation effectiveness data to be collected. For COVID-19 vaccines, the EU guidance [29] indicates other changes could be accepted within this application (e.g., a new manufacturing site); although this was acceptable during the emergency phase of the pandemic (as declared by the WHO) to accommodate justified changes (e.g., those impacting supply), it is expected that in a non-emergency scenario this will remain an exception.

While new changes to the composition of COVID-19 vaccines were processed effectively, these product versions co-existed within the original MA (ancestral strain). This created complexity in relation to the product information (which contains the prescribing information and package leaflet for patients) and data repositories. For example, each variant update necessitated changes to the invented (product) name to distinguish the different products and where one was used, to the International Nonproprietary Name (INN) (the descriptor for the drug substance). The two COVID-19 vaccines, Spikevax and Comirnaty, had both applied for INNs (granted by the WHO) as descriptors for the drug substance upon initial authorisation. INNs for vaccines are not mandatory in the EU. However, if an INN is applied for, and approved, it must be used. Assignment and approval of a recommended INN usually takes > 1 year, although the WHO had instigated a fast-track process during the emergency phase of the pandemic for COVID-19 vaccine INNs (agreement within 6 weeks). The timing of approval of the INNs, which are granted by the WHO, was critical as the companies were agreeing and preparing labelling formats and packaging of manufactured product ‘at-risk’, i.e., manufacturing of batches prior to regulatory approvals in order for the company to be ready to supply the market with sufficient product for EU member states’ immunisation campaigns to avoid any potential delay to market supply. The ‘risk’ being that such batches would require rejection if the application, as submitted, were not approved.

Rapid scientific advice provided by the EMA facilitated swift approval for the initial COVID-19 vaccine MAs and extended to necessary regulatory approvals for market supply and scale up [30]. There was early engagement with the marketing authorisation holders (MAHs) via ETF meetings/EMA (rapid) scientific advice [31], to discuss the studies and data that would be required to support the strain update. These early interactions occurred even before regulatory consensus on the strain choice(s) was available and, once a decision on strain change was taken, specific strain-related discussions were held. This type of early engagement was instrumental to achieving suitable market supply, in view of the large and complex data sets involved. Companies who sought feedback on their strains in development and key areas of concern [9] benefitted from a more streamlined path to approval.

Timelines proved challenging for MAHs to complete the necessary data package for regulatory approval while in parallel they were manufacturing ‘at risk’ starting in September/October. In the context of the emergency situation in 2022, EU regulators exceptionally accepted CMC data packages on a ‘rolling basis’ prior to submission of the formal variation application, i.e., review of packages of data as they become available from ongoing studies before the actual start of the regulatory procedure.

**Figure 2:**
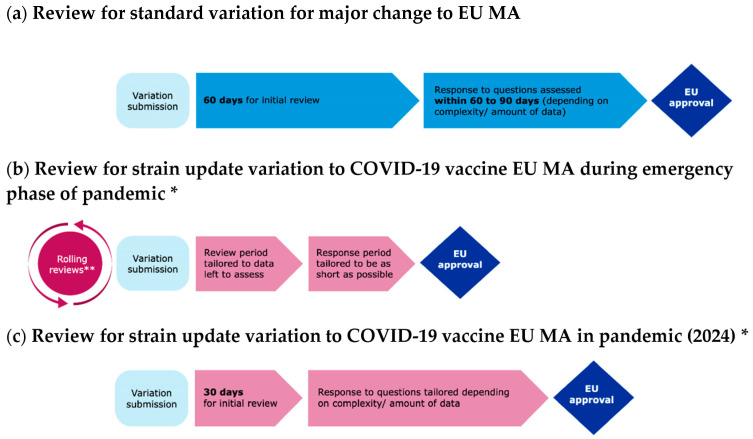
Regulatory review processes used for major changes to EU COVID-19 vaccine marketing authorisations (MAs). * Indicative estimates; eventual timelines dependent on quality of initial submission and responses to questions during the procedure. ** RR = Rolling review, review of packages of data as they become available from ongoing studies before the actual start of the regulatory procedure.

Although the 2023 variant updates were expected by companies early in the year, the actual recommendation from the WHO technical advisory group on COVID-19 vaccine composition was only made on 18 May 2023 [32]. In parallel, the WHO declared the end of the emergency situation in May 2023. This meant that the rolling review—being too resource intensive—was no longer possible for variant updates and the review process was moved to a standard review process [33]. Type II variation timetables were adapted in consideration of the autumn vaccination roll-out and the first mRNA vaccine update was completed by the end of August (for Comirnaty XBB.1.5). The recombinant protein vaccine, Nuvaxovid (XBB.1.5), received an authorisation for its update by October 2023, making it the first update to a non-mRNA COVID-19 vaccine by the EMA. Figure 2 depicts the regulatory processes used for major changes to an EU MA in comparison to the regulatory processes used for the COVID-19 vaccine strain updates during emergency and non-emergency phases.

Considerable experience has now been generated with COVID-19 vaccine variant updates to allow for advanced planning and a streamlined regulatory process. It is expected that manufacturing commitments and general manufacturing changes unrelated to the strain update are filed and completed prior to strain update procedures.

Another important element for market supply of variant vaccines is timely Official Control Authority Batch Release (OCABR) [34] testing of each batch of centrally approved COVID-19 vaccine onto the EU market. This is an extra control step routinely applied in the EU/EEA for each batch of human vaccines approved via the EMA prior to placing it on the market. The Official Medicines Control Laboratory (OMCL) performs independent testing for some key parameters and must declare that the batch in question complies with the approved specifications laid down in the marketing authorisation and in the relevant monographs of the European Pharmacopoeia. MAHs need to liaise closely with the relevant Official Medicines Control Laboratory (OMCL) to ensure that necessary tests/adaptations (e.g., to purity and content/potency) which take account of the strain-specific features of the adapted strain are made early on, to permit independent authority release of the new variant product batches.

## 4. CMC Quality Scientific Considerations for COVID-19 Vaccine Updates

### 4.1. General Considerations and Prior Knowledge

The CMC (chemistry, manufacturing, and controls) section of the application for MA concerns the design, development, and consistent manufacture of a specified medicinal product of good quality. CMC is led both by clinical needs (intended use in patients, dose and delivery of the product, etc.), which impact the process design, and also by the characteristics of the molecule itself, which affects how the molecule can be manufactured, isolated, purified, and formulated at commercial scale in quantities sufficient to address the market needs. Unlike molecules which are chemically synthesised, most vaccines are complex biological medicines made from living organisms, which are naturally variable. Thus, the active substance in the final biological medicine can have an inherent degree of minor variability (‘microheterogeneity’). This minor variability must fall within an acceptable range to ensure consistent safety and efficacy. As a consequence, a biological medicinal product can comprise a number of product variants and process-related impurities (e.g., trace amounts of impurities derived from the use of cells or materials used in manufacture). The processes must therefore be carefully controlled. Characterisation of the biological medicinal product includes a range of tests to establish the physicochemical, purity/impurity, and biological activity profiles.

The development program of the biological product needs to be carefully constructed to yield a product with defined key critical quality attributes, i.e., properties that are controlled to achieve the desired product quality. It also needs to ensure that the potential impact of the inherent variability of the biological production process on safety and efficacy are considered and suitably controlled (e.g., post-transcriptional modifications to a protein that could cause a lack of efficacy or immunogenicity). This information is used to establish appropriate quality control release specifications for each batch of product. The quality of a biological final product is assured by using knowledge of the production process to establish a combination of analytical testing *and* a pre-defined set of manufacturing process parameters and controls which have been validated to demonstrate that product of acceptable quality can be consistently manufactured (control strategy). The release specifications are chosen to include appropriate tests to monitor consistent pharmaceutical quality (e.g., tests for absence of adventitious agents) as well as clinically-related parameters, e.g., product potency (biological activity). Indeed, in addition to the MAH’s testing, as indicated earlier, each batch of COVID-19 vaccine cannot be marketed in the EU unless accompanied by an Official Control Authority Batch Release Certificate from independent testing [35].

Due to their complexity, even small changes can have a disproportionate impact on a biological product’s quality. During development of the medicinal product, changes such as upscaling manufacture are often necessary. For a biological product, comparability evaluation during development is then required to link process versions used in non-clinical and clinical studies to the product used in pivotal clinical trials and the final commercial product. After approval, further changes to the product or process have to be shown to be equivalent to the product shown to be efficacious in clinical trials. Comprehensive comparability studies (which are more extensive than routine release testing alone) are used to demonstrate that the ‘changed product’ is highly similar to the original and that “the existing knowledge is sufficiently predictive to ensure that any differences in quality attributes have no adverse impact upon safety or efficacy of the drug product” [36].

Post-approval changes are necessary for all medicinal products, including biological products. MA holders (MAHs) submit post-authorisation changes mainly to improve manufacturing efficiency and quality control of authorised products and to take account of technical and scientific progress, as required by legislation.

For all medicinal products, there are a number of guidelines and mandatory requirements that apply to development and manufacture. The EMA publishes its own scientific guidelines to aid medicinal product developers. It is also an active participant in International Council for Harmonisation (ICH) activities [37]. Once an ICH guideline has been finalised, it can be implemented as a European guideline. Additionally, in the EU, the European Pharmacopoeia [38] is a reference work for the quality control of medicines, and the official standards published within provide a legal and scientific basis for quality control during the development, production, and marketing processes. Finally, the EMA coordinates inspections to verify compliance with good manufacturing practice (GMP) standards. GMP describes the minimum standards that a medicines manufacturer must meet in their production processes, i.e., the relevant standards for activities at the manufacturing site where the product is produced [39].

Usually, a sequential approach to CMC development is taken, in the context of ongoing clinical development of a medicinal product, i.e., the step-by-step development of the medicinal product manufacturing process from the initial animal studies to early clinical studies and through the phase III clinical studies. This process usually occurs over many years and, once all data are available, an MA is submitted for regulatory review. However, during the emergency phase of the COVID-19 pandemic, there was a need to promptly develop new vaccines at an unprecedented scale. This required different, more flexible approaches for CMC, comprising regulatory data review on an ongoing basis, whilst still maintaining agreed quality standards; that is, using newer rapid read-out analytical methods. This development was conducted in parallel to the clinical development, rather than stepwise, to ensure that, once favourable clinical data supporting approval were available, the CMC package was also ready, aiming to avoid bottlenecks to patient supply [40].

For initial COVID-19 vaccine MAs, the EMA proactively supported planning of the MA via early engagement through pre-submission interactions with MAHs and provision of the EMA scientific advice via the ETF [41]. This increased predictability of the outcome and saved time during dossier preparation and review as key CMC issues were identified in advance. Similarly, this support was provided for the variant updates during the emergency phase of the pandemic.

Previous manufacturing experience, or so-called prior knowledge [42] from similar types of medicinal products, serves as an ‘acceleration-enabler’ in vaccine approvals, where the relevance of the prior knowledge has been demonstrated and justified [43]. There are also product-specific data and site specificities, etc., for the different presentations which need to be carefully maintained to ensure accurate lifecycle management.

The original COVID-19 vaccine MA made use of data derived from large numbers of batches manufactured in view of the anticipated high demand, which allowed CMC requirements to be met even under accelerated approval timelines [31]. Extrapolation of data from the ancestral vaccine with minimal changes to the new variant version has been a useful application of prior knowledge (e.g., to support comparability during changes to the drug substance and drug product manufacturing process, and to control strategy and specification setting). As an example, when evaluating drug product stability of related variant vaccines, data from representative batches of parental/previous strains were used to support the shelf-life claim where relevant. Trends in stability data have also been extrapolated using predictive stability models when sufficient prior knowledge of the previous strains was available. However, the use of knowledge from previous versions to support a change for a COVID-19 vaccine (e.g., mRNA vaccine) does not yet constitute an established manufacturing platform with general applicability to all mRNA vaccines. Therefore, prior knowledge can inform development of a similar type of vaccine to those that have been previously authorised, but companies need evidence to demonstrate the relevance of the prior knowledge for that specific product.

### 4.2. EMA CMC Guidance

The 2021 EMA reflection paper [44] acknowledged that the CMC data requirements for variant updates largely depend on the manufacturing technology used. Specific aspects were highlighted as follows:

- Changes to starting materials (e.g., DNA template, virus seed); strain specific changes to the control strategy whilst expecting that reliance on the parental vaccine control strategy (prior knowledge) should largely be possible.
- Testing of critical quality attributes (e.g., purity, content) to demonstrate compliance with the registered specifications (with any changes to the registered specifications requiring adequate scientific and/or clinical justification).
- Requirements for the demonstration of manufacturing consistency (e.g., by drug substance and drug product characterisation, in-process control results, and batch analyses).
- An expectation that shelf life might be extrapolated from the existing body of data on the parental vaccine (upon demonstration of the suitability of the drug substance and drug product registered shelf life with confirmatory real-time stability data still needing to be provided post-approval).

For multivalent products, assurance of the quality of the individual drug substances at release and up to the end of shelf life is expected (e.g., the control of relative amounts of the variants, total level of impurities, and the validity of the analytical procedures when testing multivalent products). Changes to the control strategy and release specification to reflect the additional variants, as well as adaptations in the formulation can be anticipated and should be supported by pharmaceutical development studies. Batch analysis and process validation data requirements are also different when moving from a monovalent parent vaccine to a multivalent variant vaccine, since the prior knowledge accumulated has been on the basis of the monovalent product.

### 4.3. Experience of Strain Updates and Discussions for mRNA Vaccines

Planning for the BA.1 COVID-19 update was complicated by the uncertainty of the need for a monovalent or bivalent presentation (to include the ancestral strain), and considerations of which vaccines would be needed by global public health authorities in charge of vaccination campaigns. MAHs needed time to develop suitable bivalent presentations and plan for the potential scenario of parallel supply of both bivalent/monovalent presentations across different markets. Additional sites were needed to accommodate such increased complexity in supply. These challenges highlight the importance of an aligned global ‘switch’ to assure timely supply globally.

The first BA.1 and BA.4-5 updates for the COVID-19 mRNA vaccines made use of the same formulation, control strategy, manufacturing process (aside from adaptation to bivalent manufacture), and inclusion of registered manufacturing sites for the drug product, therefore assessment was generally streamlined. Changes to the drug substance were largely limited to the nucleotide sequence of the coding region only. In few cases, changes were proposed to the non-coding region; these still required justification and demonstration that there was no impact to protein expression or functional mRNA half-life.

This approach facilitated the use of drug substance and product data supplemented by comparability of release and characterisation data of the new strain compared to historical data (previous strain). In case of major changes to the manufacturing process (e.g., addition of interim hold times affecting the release test sampling) demonstration of comparability required substantial historical data from all authorised sites with statistically justified comparability criteria to confirm that the quality of the batches was not affected by an additional subsequent hold time. Where changes were minimised, process validation requirements, i.e., demonstrated ability to consistently manufacture product of suitable quality, could be appropriately adjusted based on the comparability of the manufacturing process to the ancestral strain. For bivalent products, generally, specifications for identity were updated and tests to permit relative content limits and mRNA expression of both bivalent strains were added.

The impact of the adapted mRNA sequence on the expression of the intended protein was expected to be characterised by generating protein expression data of both variants for the bivalent products. Substantial changes to analytical methods required additional characterisation/comparability data to support that specifications were clinically justified. Results from mixing studies and characterisation data were required to support the change from monovalent to bivalent presentations.

During the public health emergency, in cases where comparability had been demonstrated between clinical and commercial-scale drug products and between manufacturing sites, adapted requirements for batch analysis data were accepted for variant vaccines, sometimes with requests for data provision post-approval.

For shelf-life determination, real-time stability data were limited at time of approval of the variant vaccine, as was the case for the approval of the original vaccine. However, extrapolation of the shelf-life was made possible based on comparability to the parent vaccine and related variant products for which a greater stability data set was available than at initial authorisation. Accelerated stability data, a post-approval stability protocol for drug substances, product for each variant, and a requirement to continue the initiated real-time stability studies were expected in each case. Where available and justified, stability models generated using additional variant mRNAs were used to incorporate sequence differences into the statistical model.

Extensions of shelf-life of the authorised vaccines were also filed based on additional stability data. Successful evaluation of these submissions led to retrospective shelf-life extensions of product already released onto the market; ‘alternative’ storage conditions, where specific schedules of storage times at different temperatures, were also provided for Spikevax [45].

At the time of the Omicron updates, experience allowed for greater reliance on data from previous variants across the CMC package for the COVID-19 mRNA vaccines. As the epidemiological situation changed over the pandemic years, many EU governments moved away from mass immunisation campaigns. This resulted in the need for single-dose presentations to be added to the MAs of approved COVID-19 vaccines. Supply chains needed to be adapted, and manufacturing sites added or removed accordingly. Careful planning of the necessary sites with respect to GMP and expected data requirements (e.g., process validation, helped avoid delays to finalisation).

### 4.4. Experience of Strain Updates/Discussions for Recombinant Vaccines

Compared to mRNA vaccines, the experience of the recombinant COVID-19 variant update progressed thus far proved to be more complex since more substantial changes are expected to manufacture these vaccines (e.g., change of cell banks with the new construct).

Changes to the recombinant protein necessary to adapt to the variant strain resulted in more structural differences relative to the approved strain. These changes might translate into different physicochemical properties and molecular size rendering comparability demonstration more complex with the need to produce more batches to support this evaluation. Confirmation of product specific features (characterisation of the new strain) was provided. These differences also impacted extrapolation of stability data.

For recombinant vaccines, comparability demonstration of the new strain compared to historical data from the previous strain also requires comparison of degradation profiles in stressed stability studies. Adaptation of a wider number of tests in addition to identity and potency across the drug substance/drug product level may be required, even if changes to the drug substance and drug product manufacturing processes are minimised and the formulation remains unchanged.

For recombinant vaccines in particular, there is a need to minimise deviations from the established manufacturing approach in order to make use of data from earlier product versions to support the new variant. The product development data package should be supported with robust characterisation data to better understand the impact of potential changes to the process and antigen. Our experience showed that strain-independent changes included within the recombinant vaccine variant update stretched assessment timelines but could be achieved. In our opinion, investment in the streamlining of manufacturing processes, particularly for these types of products, will facilitate faster and easier strain updates.

## 5. Discussion, Conclusions, and Future Directions

After authorisation of the original COVID-19 vaccines with the ancestral strain, a mechanism was needed to adapt the vaccines targeting the original SARS-CoV-2 strain to be more effective against the emerging circulating variants. For SARS-CoV-2, the pace at which variants emerge challenges the technical feasibility to make an adapted vaccine more closely matching the circulating strains available in a suitable timeframe and in sufficient numbers of doses. Thus, the approach currently aims at compositional updates intending to broaden immunity against relevant circulating SARS-CoV-2 variants.

Despite the current absence of a clear-cut seasonal spread for COVID-19, the regulatory approach adopted by the EU, thus far, has been a pragmatic approach, following a pathway similar to that of seasonal influenza. For COVID-19, the regulatory approach currently requires CMC and non-clinical data, as well as demonstrating that updated vaccines induce an immune response that can predict clinical efficacy and safety in humans.

The decisions on choice of strain are based on real-world evidence on the effectiveness of previously used vaccine strains, incorporating data on virus variant epidemiology and evolution, animal and human studies in cross-neutralisation elicited by previous vaccines against emerging variants, and animal studies of new-variant adapted vaccine candidates [4]. The pragmatic approach taken on choice of strain is broadly aligned with other global regulators and, thus far, provides protection against the worst outcomes (death and hospitalisation) of newer SARS-CoV-2 strains, as evidenced by clinical studies with the approved BA.1 and BA.4-5 [3], four bivalent vaccines, and with the adapted XBB vaccines [5,6,7,8,9].

Our experience shows that the key areas of focus for CMC submission packages were starting materials, testing of critical quality attributes, characterisation, limiting changes to release specifications, demonstration of manufacturing consistency, and justification for the proposed shelf-life. The EU experience of variant updates of COVID-19 vaccines was obtained primarily with mRNA-based vaccines. However, in our view it is important to have available vaccines with different technologies in order to provide alternatives to fulfil the needs of public health authorities and citizens. Resilience/supply chain considerations and global availability of different product types combined with public expectations for a broad choice of vaccines are also factors supporting development and authorisation of different vaccine types.

The mRNA manufacturing platforms lend themselves well to rapid adaptations of the vaccine. Changes to the drug substance were often largely limited to the nucleotide sequence which facilitated comparability demonstration to previously approved versions of the vaccines. Similar manufacturing processes also enabled timely additions of novel manufacturing sites for the drug product.

For the recombinant protein COVID-19 vaccine submission, changes to the vaccine variant included changes as discussed above. The MAH could rely to a lesser extent on an earlier product version but compensated for this with additional manufacturing verification batches. The experience indicated that investment in the streamlining of manufacturing processes and increased product understanding to better predict the impact of adaptations on the product would facilitate more straightforward future strain updates, thereby safeguarding availability of different COVID-19 vaccine types.

Prior knowledge has been useful in developing new variant vaccines, and there is value in using that knowledge to inform development of similar types of products if companies are able to collate and present data to justify its relevance for a specific product.

As for initial MA approval [30], our recommendations for the need for a continuous early regulator–developer engagement hold true and have been essential in ensuring that developers are made aware of regulatory requirements for the specific development as early as possible so that any required data to support the application can be generated.

A global consensus on when periodic recommendations for strain changes are made will facilitate greater predictability and efficiency for companies and regulators if, and when, the evolution of the virus allows. This is indeed the case for seasonal influenza updates, which allows developers, regulators, and public health bodies to more efficiently plan and target their activities to ensure timely product availability. For influenza, the EU strain recommendations for the forthcoming season usually take place in March and follow the WHO recommendations (usually in February). All regulatory influenza updates for the new strains are finalised by August, at the latest, ensuring availability of product for autumn immunisation campaigns. Currently, the EU influenza regulatory framework accommodates different types of vaccines, and it is therefore anticipated that different vaccine types could be accommodated in a potential parallel seasonal framework for COVID. It is, however, acknowledged that there are decades of experience with adaption of the influenza vaccine, whereas experience for COVID vaccines is very limited. The influenza framework consequently offers an example of the viability of different vaccine types to increase product choice for patients and health care professionals. Variant updates for COVID vaccines are likely to be more predictable as experience grows, and if a seasonal, globally-aligned mechanism for adaptation emerges.

The International Coalition of Medicines Regulatory Authorities (ICMRA) [10] provides a forum to support COVID-19 strategic coordination and foster international cooperation among global medicine regulatory authorities to achieve greater global alignment and to facilitate global approvals in the future. If data requirements for variant updates were standardised across more regions for different vaccine types, developers would increasingly be able to more rapidly supply more global markets with adapted vaccines. Aligned requirements would also provide better support to the decision-making process of all regulatory regions, especially smaller ones. An ICMRA/WHO workshop was held in February 2024 with this aim [46].

Additionally, the European Commission has published a new Directive and Regulation [47] which will revise and replace the existing general pharmaceutical legislation in the EU. Amended provisions for COVID-19 regulatory procedures, along with the use of prior knowledge and platform approaches may feature in the final text. In the context of the COVID-19 emergency, rolling reviews were essential in accelerating the pace of approvals and formal legal provisions; while unsustainable routinely due to finite resources, these rolling reviews will be critical in the regulatory response for future outbreaks.

Much experience has been gained collectively by all stakeholders to ensure provision of adapted COVID-19 vaccines to patients. For CMC, the virus evolution still poses a challenge to develop and market adapted vaccines which aim to enhance vaccine-induced immune responses to circulating SARS-CoV-2 variants once these strains emerge. Optimisation of vaccine manufacturing processes by developers and maintenance of continuous engagement between stakeholders will lead to more predictable outcomes for different COVID-19 vaccine types. Building even stronger ties across regions will reduce time for developers when preparing their data packages and for regulators to achieve a more aligned view on those data. Data digitalisation initiatives will also permit better management of product lifecycle changes and facilitate global alignment and data sharing. From a CMC perspective, we are continuously building up a wealth of knowledge on the key issues of focus when adapting different vaccine types/viruses. This is demonstrated by the 2009 H1N1 influenza pandemic vaccines which benefited from the experience gained from seasonal influenza adaptations. The 2009 influenza pandemic vaccines were themselves adapted from existing pandemic preparedness vaccines, which contained strains with pandemic potential (e.g., H5N1). We are now building up similar knowledge with the strain adaptations of different COVID-19 vaccines. This CMC knowledge, combined with the specific emergency regulatory approach described and used for COVID-19 and influenza, therefore provides a valuable blueprint for the management of vaccine adaptations in future emerging health threats. In the meantime, the EMA will continue to build on its experience of the review of the CMC parts of the COVID-19 vaccine variant application to inform more predictable, scientifically robust applications.

The views expressed in this article are the personal views of the author(s) and may not be understood or quoted as being made on behalf of or reflecting the position of the European Medicines Agency or one of its committees or working parties.

## Data Availability

No new data were created or analyzed in this study. Data sharing is not applicable to this article.

## References

[1] European Medicines Agency COVID-19 Vaccines. https://www.ema.europa.eu/en/human-regulatory/overview/public-health-threats/coronavirus-disease-covid-19/treatments-vaccines/covid-19-vaccines.

[2] European Medicines Agency Withdrawn Applications and Products. https://www.ema.europa.eu/en/human-regulatory-overview/public-health-threats/coronavirus-disease-covid-19/covid-19-public-health-emergency-international-concern-2020-23/withdrawn-applications-products.

[3] European Centre for Disease Prevention and Control ECDC-EMA Statement on Updating COVID-19 Vaccines Composition for New SARS-CoV-2 Virus Variants. https://www.ecdc.europa.eu/sites/default/files/documents/covid-19-vaccines-composition-variants-statement-ECDC-EMA_0.pdf.

[4] European Medicines Agency EMA Recommendation to Update the Antigenic Composition of Authorised COVID-19 Vaccines for 2024–2025. https://www.ema.europa.eu/en/documents/other/ema-recommendation-update-antigenic-composition-authorised-covid-19-vaccines-2024-2025_en.pdf.

[5] Andersson N.W., Thiesson E.M., Baum U., Pihlström N., Starrfelt J., Faksová K., Poukka E., Meijerink H., Ljung R., Hviid A. (2023). Comparative effectiveness of bivalent BA.4-5 and BA.1 mRNA booster vaccines among adults aged ≥50 years in Nordic countries: Nationwide cohort study. BMJ.

[6] Chae C., Kim R.K., Jang E.J., Shim J.A., Park E., Lee K.H., Hong S.L., Aziz A.B., Tadesse B.T., Marks F. (2023). Comparing the effectiveness of bivalent and monovalent COVID-19 vaccines against COVID-19 infection during the winter season of 2022-2023: A real-world retrospective observational matched cohort study in the Republic of Korea. Int. J. Infect. Dis..

[7] Wang Q., Guo Y., Bowen A., Mellis I.A., Valdez R., Gherasim C., Gordon A., Liu L., Ho D.D. (2024). XBB.1.5 monovalent mRNA vaccine booster elicits robust neutralizing antibodies against XBB subvariants and JN.1. Cell Host Microbe.

[8] Hansen C.H., Moustsen-Helms I.R., Rasmussen M., Søborg B., Ullum H., Valentiner-Branth P. (2024). Short-term effectiveness of the XBB.1.5 updated COVID-19 vaccine against hospitalisation in Denmark: A national cohort study. Lancet Infect. Dis..

[9] van Werkhoven C.H., Valk A.-W., Smagge B., de Melker H.E., Knol M.J., Hahné S.J., van den Hof S., de Gier B. (2024). Early COVID-19 vaccine effectiveness of XBB.1.5 vaccine against hospitalisation and admission to intensive care, the Netherlands, 9 October to 5 December 2023. Eurosurveillance.

[10] International Coalition of Medicines Regulatory Authorities COVID-19. https://icmra.info/drupal/en/covid-19.

[11] European Medicines Agency Emergency Task Force (ETF). https://www.ema.europa.eu/en/committees/working-parties-other-groups/emergency-task-force-etf.

[12] European Medicines Agency Comirnaty. https://www.ema.europa.eu/en/medicines/human/EPAR/comirnaty.

[13] European Medicines Agency Spikevax (Previously COVID-19 Vaccine Moderna). https://www.ema.europa.eu/en/medicines/human/EPAR/spikevax-previously-covid-19-vaccine-moderna.

[14] European Medicines Agency Vaxzevria (Previously COVID-19 Vaccine AstraZeneca). https://www.ema.europa.eu/en/medicines/human/EPAR/vaxzevria-previously-covid-19-vaccine-astrazeneca.

[15] European Medicines Agency Jcovden (Previously COVID-19 Vaccine Janssen). https://www.ema.europa.eu/en/medicines/human/EPAR/jcovden-previously-covid-19-vaccine-janssen.

[16] European Medicines Agency COVID-19 Vaccine (Inactivated, Adjuvanted) Valneva. https://www.ema.europa.eu/en/medicines/human/EPAR/covid-19-vaccine-inactivated-adjuvanted-valneva.

[17] European Medicines Agency Nuvaxovid. https://www.ema.europa.eu/en/medicines/human/EPAR/nuvaxovid.

[18] World Health Organization Tracking SARS-CoV-2 Variants. https://www.who.int/activities/tracking-SARS-CoV-2-variants.

[19] Duong D. (2021). Alpha, Beta, Delta, Gamma: What’s important to know about SARS-CoV-2 variants of concern?. Can. Med. Assoc. J..

[20] World Health Organization Weekly Epidemiological Update on COVID-19—21 December 2021. https://www.who.int/publications/m/item/weekly-epidemiological-update-on-covid-19---21-december-2021.

[21] World Health Organization Weekly Epidemiological Update on COVID-19—25 January 2022. https://www.who.int/publications/m/item/weekly-epidemiological-update-on-covid-19---25-january-2022.

[22] World Health Organization Update 80: What We Know about New COVID-19 Variants of Concern—The Latest on the Global Situation & Omicron BA.4/5. https://www.who.int/publications/m/item/update-80-covid-19-omicron-ba-4-5-update.

[23] European Medicines Agency VidPrevtyn Beta. https://www.ema.europa.eu/en/medicines/human/EPAR/vidprevtyn-beta.

[24] European Medicines Agency The Evaluation of Medicines, Step-by-Step. https://www.ema.europa.eu/en/human-regulatory-overview/marketing-authorisation/evaluation-medicines-step-step.

[25] European Medicines Agency From Laboratory to Patient: The Journey of a Medicine Assessed by EMA. https://www.ema.europa.eu/en/documents/other/laboratory-patient-journey-centrally-authorised-medicine_en.pdf.

[26] Cavaleri M., Sweeney F., Gonzalez-Quevedo R., Carr M. (2021). Shaping EU medicines regulation in the post COVID-19 Era. Lancet Reg. Health—Eur..

[27] European Medicines Agency Influenza Vaccines—Submission and Procedural Requirements—Scientific Guideline. https://www.ema.europa.eu/en/influenza-vaccines-submission-procedural-requirements-scientific-guideline.

[28] European Medicines Agency Guideline on Influenza Vaccines—Quality Module. https://www.ema.europa.eu/en/documents/scientific-guideline/guideline-influenza-vaccines-quality-module-revision-1_en.pdf.

[29] European Medicines Agency Procedural Guidance for Variant Strain(s) Update to Vaccines Intended for Protection Against Human Coronavirus. https://www.ema.europa.eu/en/documents/regulatory-procedural-guideline/procedural-guidance-variant-strains-update-vaccines-intended-protection-against-human-coronavirus_en.pdf.

[30] Shivji R., Conocchia R., Korakianiti E., Jekerle V. (2022). Considerations for the chemistry, manufacturing and Controls (CMC)—quality package for COVID-19 vaccines- interim lessons learnt by the European medicines Agency (EMA). Vaccine.

[31] European Medicines Agency COVID-19 Guidance: Research and Development. https://www.ema.europa.eu/en/human-regulatory-overview/public-health-threats/coronavirus-disease-covid-19/covid-19-public-health-emergency-international-concern-2020-23/guidance-medicine-developers-and-other-stakeholders-covid-19/covid-19-guidance-research-development.

[32] World Health Organization Statement on the Antigen Composition of COVID-19 Vaccines. https://www.who.int/news/item/18-05-2023-statement-on-the-antigen-composition-of-covid-19-vaccines.

[33] European Medicines Agency Phasing out of Extraordinary COVID-19 Regulatory Flexibilities. https://www.ema.europa.eu/en/news/phasing-out-extraordinary-covid-19-regulatory-flexibilities.

[34] European Directorate for the Quality of Medicines & HealthCare Human OCABR Guidelines. https://www.edqm.eu/en/omcl/human-ocabr-guidelines.

[35] European Directorate for the Quality of Medicines and HealthCare Human Biologicals (OCABR). https://www.edqm.eu/en/omcl/human-biologicals-ocabr-.

[36] International Council for Harmonisation of Technical Requirements for Pharmaceuticals for Human Use Comparability of Biotechnological/Biological Products Subject to Changes in Their Manufacturing Process Q5E. https://database.ich.org/sites/default/files/Q5E%20Guideline.pdf.

[37] International Council for Harmonisation of Technical Requirements for Pharmaceuticals for Human Use. https://www.ich.org/.

[38] European Directorate for the Quality of Medicines & HealthCare European Pharmacopoeia (Ph. Eur.). https://www.edqm.eu/en/european-pharmacopoeia.

[39] European Medicines Agency Good Manufacturing Practice. https://www.ema.europa.eu/en/human-regulatory-overview/research-development/compliance-research-development/good-manufacturing-practice.

[40] Popkin M.E., Goese M., Wilkinson D., Finnie S., Flanagan T., Campa C., Clinch A., Teasdale A., Lennard A., Cook G. (2022). Chemistry Manufacturing and Controls Development, Industry Reflections on Manufacture, and Supply of Pandemic Therapies and Vaccines. Aaps J..

[41] European Medicines Agency Scientific Advice and Protocol Assistance. https://www.ema.europa.eu/en/human-regulatory-overview/research-development/scientific-advice-protocol-assistance.

[42] European Medicines Agency Toolbox Guidance on Scientific Elements and Regulatory Tools to Support Quality Data Packages for PRIME and Certain Marketing Authorisation Applications Targeting an Unmet Medical Need. https://www.ema.europa.eu/en/toolbox-guidance-scientific-elements-and-regulatory-tools-support-quality-data-packages-prime-and-certain-marketing-authorisation-applications-targeting-unmet-medical-need-scientific-guideline.

[43] Castellanos M.M., Gressard H., Li X., Magagnoli C., Moriconi A., Stranges D., Strodiot L., Tello Soto M., Zwierzyna M., Campa C. (2023). CMC Strategies and Advanced Technologies for Vaccine Development to Boost Acceleration and Pandemic Preparedness. Vaccines.

[44] European Medicines Agency Reflection Paper on the Regulatory Requirements for Vaccines Intended to Provide Protection against Variant Strain(s) of SARS-CoV-2. https://www.ema.europa.eu/en/documents/scientific-guideline/reflection-paper-regulatory-requirements-vaccines-intended-provide-protection-against-variant-strains-sars-cov-2_en.pdf.

[45] European Medicines Agency Spikevax Product Information. https://www.ema.europa.eu/en/documents/product-information/spikevax-previously-covid-19-vaccine-moderna-epar-product-information_en.pdf.

[46] International Coalition of Medicines Regulatory Authorities Report from the ICMRA/WHO Workshop on: Global Perspectives on COVID-19 Vaccines Strain Update. Alignment on Timing and Data Requirements. https://icmra.info/drupal/covid-19/26_27february2024.

[47] European Commission A Pharmaceutical Strategy for Europe. https://health.ec.europa.eu/medicinal-products/pharmaceutical-strategy-europe_en.

